# SAMHD1-dependent retroviral control and escape in mice

**DOI:** 10.1186/1742-4690-10-S1-P70

**Published:** 2013-09-19

**Authors:** Jan Rehwinkel, Jonathan Maelfait, Rachel Rigby, Anne Bridgeman, Caetano Reis e Sousa

**Affiliations:** 1 Medical Research Council Human Immunology Unit, Radcliffe Department of Medicine, Medical Research Council Weatherall Institute of Molecular Medicine, University of Oxford, Oxford, UK; 2 Immunobiology Laboratory, Cancer Research UK London Research Institute, London, UK

## 

SAMHD1 is a host restriction factor for human immunodeficiency virus 1 in cultured human cells. SAMHD1 mutations cause autoimmune Aicardi-Goutières syndrome and are found in cancers including chronic lymphocytic leukemia. SAMHD1 is a triphosphohydrolase that depletes the cellular pool of deoxynucleoside triphosphates, thereby preventing reverse transcription of retroviral genomes. However, *in vivo* evidence for SAMHD1’s antiviral activity has been lacking. We generated *Samhd1* null mice, which do not develop autoimmune disease despite displaying a type I interferon signature in spleen, macrophages and fibroblasts. *Samhd1^-/-^* cells have elevated dNTP levels but, surprisingly, SAMHD1-deficiency did not lead to increased infection with VSV-G-pseudotyped HIV-1 vectors. The lack of restriction is likely attributable to the fact that dNTP concentrations in SAMHD1 -sufficient mouse cells are higher than the K_M_ of HIV-1 reverse transcriptase. Consistent with this notion, an HIV-1 vector mutant bearing a reverse transcriptase with lower affinity for dNTPs was sensitive to SAMHD1-dependent restriction in cultured cells and in mice. This shows that SAMHD1 can restrict lentiviruses *in vivo* and that nucleotide starvation is an evolutionarily conserved antiviral mechanism.

